# *Bacillus* Spp.: Efficient Biotic Strategy to Control Postharvest Diseases of Fruits and Vegetables

**DOI:** 10.3390/plants8040097

**Published:** 2019-04-12

**Authors:** Oksana Lastochkina, Maryam Seifikalhor, Sasan Aliniaeifard, Andrey Baymiev, Ludmila Pusenkova, Svetlana Garipova, Darya Kulabuhova, Igor Maksimov

**Affiliations:** 1 Bashkir Research Institute of Agriculture, Ufa Federal Research Centre of the Russian Academy of Sciences, 450059 Ufa, Russia; L.Pusenkova@mail.ru (L.P.); garipovasvetlana@gmail.com (S.G.); dariya.greatfire@mail.ru (D.K.); 2 Institute of Biochemistry and Genetics, Ufa Federal Research Centre of the Russian Academy of Sciences, 450054 Ufa, Russia; baymiev@anrb.ru (A.B.); igor.mak2011@yandex.ru (I.M.); 3 Department of Plant Biology, Center of Excellence in Phylogeny of Living Organisms in Iran, School of Biology, College of Science, University of Tehran, Tehran 14155, Iran; mskalhor@ut.ac.ir; 4 Department of Horticulture, College of Aburaihan, University of Tehran, Pakdasht, Tehran 3391653775, Iran; aliniaeifard@ut.ac.ir; 5 Department of Biology, Bashkir State University, 450076 Ufa, Russia

**Keywords:** *Bacillus* spp., endophytic *Bacillus subtilis*, biocontrol, postharvest diseases, food losses, storage, fruits/vegetables

## Abstract

Postharvest diseases significantly reduce the shelf-life of harvested fruits/vegetables worldwide. *Bacillus* spp. are considered to be an eco-friendly and bio-safe alternative to traditional chemical fungicides/bactericides due to their intrinsic ability to induce native anti-stress pathways in plants. This review compiles information from multiple scientific databases (Scopus, ScienceDirect, GoogleScholar, ResearchGate, etc.) using the keywords “postharvest diseases”, “*Bacillus*”, “*Bacillus subtilis*”, “biocontrol”, “storage”, “losses”, and “fruits/vegetables”. To date, numerous examples of successful *Bacillus* spp. application in controlling various postharvest-emerged pathogens of different fruits/vegetables during handling, transportation, and storage have been described in the literature. The mechanism/s of such action is/are still largely unknown; however, it is suggested that they include: i) competition for space/nutrients with pathogens; ii) production of various bio-active substances with antibiotic activity and cell wall-degrading compounds; and iii) induction of systemic resistance. With that, *Bacillus* efficiency may depend on various factors including strain characteristics (epiphytes or endophytes), application methods (before or after harvest/storage), type of pathogens/hosts, etc. Endophytic *B. subtilis*-based products can be more effective because they colonize internal plant tissues and are less dependent on external environmental factors while protecting cells inside. Nevertheless, the mechanism/s of *Bacillus* action on harvested fruits/vegetables is largely unknown and requires further detailed investigations to fully realize their potential in agricultural/food industries.

## 1. Introduction

Fresh fruits and vegetables encounter disease between harvest and consumption, resulting in significant food waste and economic losses. According to the Food and Agriculture Organization [1] about 45% of harvested fruits, vegetables, roots, and tubers are lost. Most of this loss occurs during storage due to pest and pathogen infestation (bacteria, fungi, and insects), unfavorable environmental conditions (rain, humidity, frost, and heat), water loss, saccharification, and sprouting [2,3]. Traditionally, chemical fungicides and/or food preservatives are used to control postharvest decay. However, exposure to these chemicals is, in many cases, hazardous to humans, animals, and the environment [4]. Due to the toxicological risk of residual chemicals in food products, their application in the postharvest period has been limited to a few registered chemicals and is completely prohibited in some European countries [5]. The increasing relevance of food and environmental problems, as well as growing demand for energy conservation through natural “green” technologies and organic products, would make it highly desirable to have an approach to the reduction of postharvest food losses that is novel, efficient, environmentally friendly, and bio-safe. Biological products based on beneficial strains, such as plant growth-promoting bacteria (PGPB), could be considered as a research-led alternative to synthetic fungicides and/or food preservatives in the control of postharvest diseases. These products establish various physiological changes in host plant metabolism, leading to systemic resistance and prolonged shelf-life without causing adverse effects on plants, humans, or the environment [6,7,8].

PGPB are a group of non-pathogenic beneficial bacteria that can directly and/or indirectly promote plant growth, disease resistance, and abiotic stress tolerance. They may live autonomously in the soil or colonize the rhizosphere, phyllosphere (epiphytes), and plants’ interior tissues (endophytes) [6,9,10,11,12]. A particularly interesting PGPB, belonging to the genus *Bacillus* spp. viz. *B. subtilis*, is one of the most attractive agents for the development of natural plant protection products, as recommended by United States Food and Drug Administration. *Bacillus* spp. are generally recognized as safe microorganisms for application in the food industry. *Bacillus* spp. occupy the same niche as many pathogens and have the capacity to produce a wide range of bio-active substances with antibiotic activity. These substances induce various physiological features in host plant metabolism without causing adverse effects on the environment and human health [8,13]. Furthermore, *Bacillus* spp. (i.e., *B. subtilis*), produce endospores resistant to dynamic physical and chemical treatments, such as heat, desiccation, organic solvents, and UV irradiation, which therefore maintain their ability to trigger defense responses in host plants, even under unfavorable conditions [14,15,16]. This makes it capable of easy-formulation and storage of *Bacillus*-based biological products and serves as a potent bio-active component against pathogens [17,18,19,20,21]. Nowadays, the protective effect of *Bacillus* strains on various plant species against a wide range of biotic (pathogens, pests) [22,23,24,25,26,27] and abiotic (drought, salinity, extreme temperatures, toxic metals, etc.) stresses [6,11,12,23,28,29,30] is well documented in the literature. The beneficial effects of *Bacillus* can be attributed to the synthesis of a wide range of biologically active compounds including antibiotics, siderophores, lipopeptides (LPs), enzymes, 1-aminocyclopropane-1-carboxylate (ACC) deaminase, and exopolysaccharides. Furthermore, *Bacillus* spp. are known to affect regulation of phytohormone biosynthesis pathways, modulate ethylene levels in plants, and influence the emission of volatile organic compounds (VOCs) and the launch of host plants’ systemic resistance/tolerance [7,9,11,14,24,27,31,32,33].

The use of *Bacillus* spp., including *B. subtilis*, for biocontrol of various postharvest diseases, in a wide range of fresh-cut fruit/vegetables during handling, transportation, and storage, has been frequently reported [7,33,34,35]. For example, the ability of *B. subtilis* to suppress the development of postharvest pathogens causing grey mold (*Botrytis cinerea* and *B. mali*) has been demonstrated in strawberry, pear, apple, and tomato [36,37,38,39,40,41,42]. More in-depth studies suggest that microbial antagonists from the *Bacillus* genus possess substantial potential to increase vegetables/fruit sets, quality, postharvest disease resistance, and tolerance under temperature fluctuations, mechanical injury associated with loading of product for transportation, unloading, packaging, and storage [33,43]. However, despite the well-studied role for *Bacillus* spp. in plant growth, development, and health under both normal and stressed conditions, their role in controlling postharvest disease, and the underlying mechanisms regulating fruits/vegetables storage quality remain largely unknown.

## 2. *Bacillus* spp. Capacity to Alleviate Postharvest Losses of Fruits and Vegetables

Although the first reported microbial antagonist application was of *Trichoderma* spp. for the control of rot (*Botrytis*) in strawberry [44], the first classical operation belongs to the control of brown rot (*Lasiodiplodia theobromae*) in stone fruits by *B. subtilis* [45]. Later, new information about the positive effect of antagonistic microorganisms, including *Bacillus* spp., on postharvest physiology of various fruit/vegetables began to emerge, establishing them as enhancers of fresh fruit/vegetable resistance against a broad spectrum of postharvest diseases and unfavorable storage conditions associated with extending shelf life and maintaining nutritional qualities [33] (Table 1). For example, postharvest disease development of melon (*Cucumis melo* L.) fruit caused by *Alternaria alternata* was suppressed up to 77.2% when treated by *B. subtilis* EXWB1 prior to storage. In the presence of EXWB1 strain, *A. alternata* hyphae growth was hampered on melon fruit surfaces as well as wounded tissues. It was suggested that such a positive effect of *B. subtilis* EXWB1 on postharvest physiology of melon is connected to the ability of the EXWB1 strain to diminish the production of ethylene up to 72.3% and decrease the respiration rate of infected and non-infected melons by 26.1% and 71.9%, respectively [34]. A possible scenario for delayed senescence and rot development in melon fruit is suppressed ethylene biosynthesis caused by *B. subtilis* EXWB1. Likewise, it was revealed that treatment of fruit with EXWB1 contributes to maintenance of turgor pressure, titratable acidity in value close to the fresh fruit, increase of total sugar content up to 36.7%, and reduction of weight loss during storage [34]. In another study of different bacterial isolates (*B. subtilis*, *B. pumilus*, *B. cereus*, *B. megaterium*, and *Agrobacterium radiobacter*), treatment by strains of *B. subtilis* and *A. radiobacter* were most effective in controlling postharvest citrus fruit disease induced by *Penicillium digitatum* [46]. It has been consistently demonstrated that *B. pumilus* and *B. amyloliquefaciens* effectively suppress the development of grey mold disease of pears and tomatoes caused by *Botrytis cinerea* [38]. *Bacillus* strains (*B. pumilus* B19, *B. subtilis* 1J, *B. crerus* B16, *B. subtilis* B11, and *B. cereus* B17) controlled grey mold of apple caused by *Botrytis mali* [41], in which the inhibitory effect varied from 13.6 to 74% in the dual culture samples; 12.3 to 87% in the cell-free metabolite tests; and 11 to 53% in the volatile experiment [47]. Moreover, the efficacy of *B. subtilis* AG1 against vine wood fungal pathogens of *Phaeoacremonium aleophilum*, *Phaeomoniella chlamydospora*, *Verticillium dahliae*, and *Botryosphaeria rhodina* has also been demonstrated [48]. The effectiveness of *B. subtilis* producing antibiotics and VOCs in suppressing the development of postharvest pathogens such as *Rhizopus stolonifer* (soft rot), *Botrytis cinerea* (gray mold), and *Colletotrichum* spp. in strawberry (*Fragaria* x *ananassa*) berries has been revealed [49]. *B. subtilis* SK1-2 strain, exhibiting high antagonistic activity against *Botryosphaeria dothidea*, *Diaporthe actinidiae*, and *Botrytis cinerea* in in vitro culture, has been introduced as an effective antagonist for the bio-control of postharvest rot in kiwifruit [35]. The strain *B. subtilis* 9407 also exhibits strong antifungal activity against *Botryosphaeria dothidea* and significantly reduces the development of ring rot of apple fruit [50]. Significant suppression by *B. subtilis* of postharvest fungal rot caused by *Aspergillus niger*, *Botryodiploidia theobromae*, and *Penicillium oxalicum* has been reported in *Dioscorea* fruit [51]. The application of *B. subtilis* strain GA1 led to a reduced level of postharvest infection in apple caused by *Botrytis cinerea* during the first 5 d following pathogen inoculation and, notably, ~80% protection over the next 10 d [39]. Inhibitory effect of *B. subtilis* treatment on both fading of the litchi fruit during storage (30 d at 5 °C), and maintenance of proper quality (total dissolved solids, ascorbic acid contents, and titratable acidity) has been revealed. Interestingly, in all treatments by *B. subtilis* bacterial cells no change in fruit taste has been reported [33]. Peaches pre-treated with *B. subtilis* CF-3 strain retained 65% of fruit quality after storage for 36 d at 10 °C, which was more than 30% higher in comparison to quality of non-treated controls [16]. Singh and Deverall (1984) reported that bacterial antagonist *B. subtilis* alleviated citrus fruit decay caused by *Penicillium digitatum* and *P. italicum*. It has been shown that *B. subtilis* H110 significantly reduced blue mold disease caused by *P. expansum* and black spot disease caused by *Alternaria alternata* on harvested apple pears (*Pyrus bretschneideri* Rehd.). It was suggested that mechanism by which *B. subtilis* H110 inhibits pathogens might be related to its ability to produce antagonistic protein and natural competition for nutrients and space [51]. An effective role for *B. subtilis* in the control of fungal rot in citrus [37] and *Monilinia fructicola* infection in peaches and cherries has also been shown [45,52]. Processing of harvested apples with suspensions of *B. subtilis* strains APEC170 and *Paenibacillus (Bacillus) polymyxa* APEC136 reduced the symptoms of anthracnose of fruits caused by the fungal pathogens of *Colletotrichum gloeosporioides* and *C. acutatum*, as well as white rot caused by *Botryosphaeria dothidea* [36]. *B. subtilis* SM21 effectively controlled rot caused by *Rhizopus stolonifer* and other pathogens including *Monilinia fructicola*, *Cephalothecium*, *Rhizoctonia*, and *Alternaria* in harvested peach fruit [53]. Co-application of *B. subtilis* and commercial wax Tag, enriched with different concentrations, significantly reduced the development of anthracnose, the avocado fruit rot complex (*Dothiorella*/*Colletotrichum*) during storage time [54]. A similar result has been obtained by immersion of fruit in water containing bacterial cells. Research on the effect of *B. subtilis* Ch-13 on induction of defense responses in potatoes against pathogens during both growing season and cold storage have indicated reduced colonization of bacterial antagonists on potato tuber surface during the storage period [55]. Application of microbial preparations for intensifying the adaptive immune response of potato tubers during cold storage is an effective approach that more than doubles the defense response in tubers. In fact, in comparison with control, *B. subtilis*-based microbial preparation increased the activity of peroxidase enzymes in potato tubers, formation of phytoalexin and ascorbic acid content by 2.4, 3.1, and 1.3 times, respectively. Treatment of banana fruit (*Musa* sp.) with the composition of bacterial strains (*Pseudomonas fluorescens* Pf1, *Bacillus* sp. EPB10, and *Bacillus* sp. EPB56) reduced the development of *Fusarium oxysporum* [56].

Of particular interest for bio-control of postharvest diseases are endophytic strains of *Bacillus* spp. due to their ability to colonize internal plant tissues and live in the same ecological niches as pathogens. This capacity allows them to survive independently from external environmental factors while conferring economically “useful” properties in host plants [32,57,58]. For example, the introduction of endophytic bacteria (*B. subtilis* 26D) into host plant tissues, before planting or during the vegetative phase, promoted plant (potato) growth, and protected plants from certain defects. These effects were maintained for a prolonged period leading to the better preservation of vegetables during storage [59]. In another study, endophytic *B. subtilis* 10-4, applied alone or in combination with natural safe signaling molecules, such as salicylic acid (SA), promoted anti-stress activity in plants [60,61]. Harvested tubers treated with *B. subtilis* 10-4 and SA were less infected by the pathogenic micromycetes *Aspergillus*, *Pennicillium*, and *Alternaria* with fully faded *Cladosporium*, *Fusarium*, and *Mucor* when compared to non-treated controls. Additionally, tubers treated with both *B. subtilis* 10-4 and *B. subtilis* 10-4 with SA retained antifungal activity even after a 30 d storage period compare to the non-treated controls, indicating the prolonged protective effect caused by *B. subtilis* treatment [60]. Interestingly, co-application of endophytic *B. subtilis* together with SA is more effective in bio-controlling potato diseases during storage than *B. subtilis* alone [59,60]. Perhaps this effect is due to the fact that SA induces plant disease resistance [61,62,63]. SA can be used as both preharvest and postharvest control strategies, giving it high commercial potential for enhancing nutritional quality and extending the shelf-life of fruit/vegetables. SA reduces chilling injury and decay, delays ripening, and enhances the health benefits of fresh fruit/vegetable consumption by enhancing disease resistance and antioxidant capacity [61]. Thus, the application of bacterial antagonists, in particular endophytic strains, either alone or in combination with other natural regulators, up-regulates the defense response in plant tissues of harvested fruits/vegetables during their time in storage. This opens a new insight into the development of effective bio-active components to extend crop longevity while maintaining quality and nutrition. However, the lack of knowledge about underlying mechanisms of the interactions within systems *Bacillus* spp.–host plant–pathogen is one of the main barriers to the commercial development of preparations based on antagonistic bacteria and their compositions.

## 3. Potential Modes of Action of Microbial Antagonists

**Figure 1:**
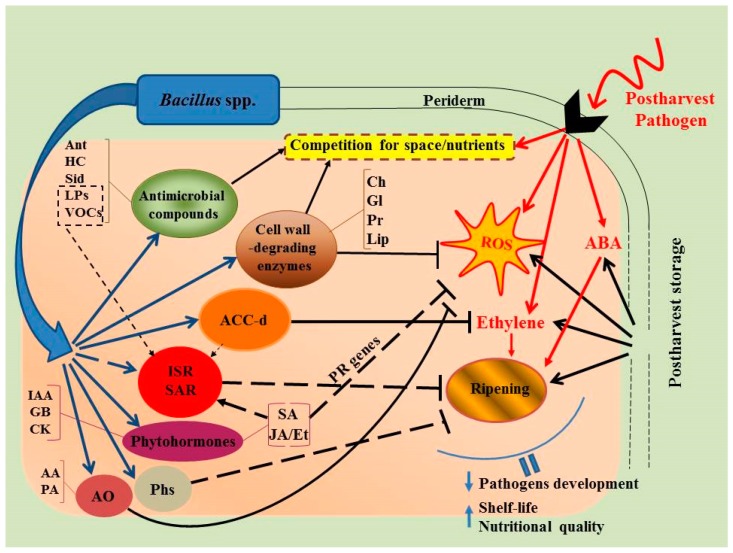
The main mechanisms of *Bacillus* action against pathogenic infections and their interaction in harvested fruits/vegetables during storage. AA—ascorbic acid, ABA—abscisic acid, ACC-*d*—1-aminocyclopropane-1-carboxylate deaminase, Ant—antibiotics, AO—antioxidants, Ch—chitinases, CK—cytokinins, Et—ethylene, GB—gibberellins, Gl—glucanases, HC—hydrogen cyanide, IAA—indole-3-acetic acid, ISR—induced systemic resistance, JA—jasmonic acid, Lip—lipases, PA—peroxidase, Pr—proteases, Phs—phytoalexins, ROS—reactive oxygen species, SA—salicylic acid, SAR—systemic acquired resistance, Sid—siderophores.

Unraveling the mechanism/s of action of microbial antagonists including *Bacillus* spp. will play a pivotal role in determining the most effective application that maximally inhibits pathogen growth and function on a harvested host plant [71]. Although the mechanisms by which *Bacillus* spp. suppresses postharvest pathogens are not yet fully understood, multiple mechanisms have been suggested including: (1) competition with pathogenic microflora for nutrients and suitable niches for colonization [23,33,64,72]; (2) production of various metabolites with antibiotic activity—antibiotics, biosurfactants, siderophores, hydrogen cyanide, etc. [49,73,74,75,76]; (3) synthesis of hydrolytic enzymes, such as chitinases, glucanases, proteases, and lipases, which can destroy pathogenic fungal cells and a number of pathogen effector compounds [53,77,78]; and (4) elicitor activity and the induction of systemic (whole host plant) resistance (ISR and SAR) [9,77,79,80] (Figure 1). The metabolism of these components are mainly regulated by host-generated hormones such as SA, ABA, JA, and ethylene, and occur due to the bacterial determinants (MAMPs, from microbe-association molecular patterns), including flagellins, lipopolysaccharides, siderophores, antibiotics, biosurfactants, and VOCs [49,57,75,79,81].

### 3.1. Competition for Space and Nutrients

Inhibition of rapid wound site colonization in fruits/vegetables triggered by antagonistic microorganisms is a crucial step in controlling postharvest decay (Figure 1). The effectiveness of antagonists mainly depends on their ability to outperform pathogens based on their capacity for rapid growth and survival under unfavorable conditions, and is strongly dependent on their initial concentration when applied on the wound site [71]. In most harvested fruits and vegetables the bio-control activity of bacterial strains is elevated by increasing their concentration as well as the reduced level of pathogens. The most effective concentration in controlling postharvest fruit/vegetable diseases is generally considered to be 10^7^–10^8^ CFU/mL [74]. As similar ecological niches exist in both endophytic and phytopathogenic microorganisms, endophytes are considered as prime candidates for bio control of phytopathogens. Due to the stable pH, proper humidity, sufficient nutrient flow, and lack of competition in the endosphere, endophytes have a significant advantage over epiphytic organisms available in the rhizosphere and phyllosphere [32,57,58].

Another significant contribution in bio-control of pathogens is competition for nutrients. In vitro studies demonstrated that bacterial inoculants take up nutrients faster than pathogens; this can lead to the inhibition of germination of pathogen spores at the wound site [82,83]. A fundamental strategy for nutrient competition is the attachment of microbial antagonists to the hyphae of a pathogen due to the fact that the antagonists feed on nutrients faster than the target pathogen, thus hampering spore germination and pathogen growth [82,84]. Nevertheless, in certain cases such as *Aureobasidium pullulans* against *Botrytis cinerea*, *Rhizopus stolonifer*, *Penicillium expansum*, and *Aspergillus niger*, which infect table grapes and *P. expansum* and *B. cinerea* on apple fruit, direct physical interaction is not required for the antagonistic activity [85]. In such circumstances antagonism does not occur via direct attachment of antagonistic microorganism to pathogen hyphae. Rather, it is highly likely that other alternative mechanisms, such as the production of a wide range of biologically active molecules, such as antibiotics, biosurfactants, siderophores, hydrogen cyanide, and hydrolases increase their advantage against pathogens as they compete for a suitable niche for colonization [76,85,86].

### 3.2. Production of Antimicrobial Compounds

Antibiotics are a heterogeneous group of low molecular weight organic compounds produced by bacteria, which suppress or diminish the growth and development of phytopathogenic microorganisms [13,14,87]. Antibiotics can cause disruption in a microorganism’s cell wall structure or membrane function, disrupt protein synthesis, and inhibit respiratory enzyme function [88]. Thus, most *Bacillus* antibiotics are active against both gram-positive and gram-negative bacteria, as well as phytopathogenic fungi such as *Aspergillus flavus*, *Alternaria solani*, *Fusarium oxysporum*, *Botryosphaeria ribis*, *Helminthosporium maydis*, *Phomopsis gossypii*, and *Colletotrichum gloeosporioides* [89]. *Bacillus* antibiotic substances break growing hyphae tips of *Sclerotinia sclerotorum* (the stimulant of sunflower white rot), *A. alternata*, *Drechlera oryrae* as soil fungi, and *F. roseum*, as well as *Puccinia graminis* (the inducer of cereals rust) [89]. It has been found that *B. subtilis* has broad suppressive properties against more than 23 types of plant pathogens in vitro due to its ability to produce a broad range of antibiotics with a wide variety of structures and activities [87]. Some *Bacillus* species may dedicate up to 8% of their genetic potential to the synthesis of a wide range of antimicrobial compounds, among which non-ribosomally synthesized LPs, lytic enzymes, and lantibiotics are suggested to be crucial for pathogen suppression [75,79,90,91,92]. According to Stein [87] in *B. subtilis* and *B. amyloliquefaciens*, ~5% and ~8% of the genomes are involved in the biosynthesis of wide range of antimicrobial compounds, respectively. In another study Ahmad and colleagues [93] reported that among 114 antagonistic genes, which are differentially expressed by *B. subtilis* (strain 330-2), about 10% were involved in antibiotic biosynthesis.

LPs synthesized by *Bacillus* spp. play pivotal roles in suppressing disease, giving them substantial status in the agriculture and biotechnology industries [49,94,95,96,97]. The molecules of the known LPs contain 4 to 16 amino acid residues; peptide chains are linear, cyclic, or mixed. Amino acid residues have an L- or D-configuration [98]. Depending on the amino acid sequence and the branches of fatty acids, microbial LPs can be divided into three main families: 1) surfactins; 2) iturins (iturin A, mycosubtilin, bacillomycin); and 3) fengycins (including pliapastatin) [15,99], which are well-studied *Bacillus* spp. cyclic LPs (CLPs) [15,39,93,98,100,101]. The lipopeptide polymyxin A from *Paenibacillus polymyxa* was discovered in 1949 and categorized in the first group [102]. In the same year Jones described the *B. subtilis* (strain IAM1213) as the most effective producer of LPs [76,102]. Since then many researchers have started detailed investigations about LPs, to distinguish them from other strains of the genus *Bacillus*, as well as actinomycetes and fungi. It was discovered that CLPs have a certain antibiotic selectivity. Therefore, if surfactin is mainly characterized by its antibacterial and antiviral properties, iturin and fungicin could be considered antifungal components [103,104]. The majority of *B. subtilis* strains originating from private or public collections have been categorized as surfactin-producers [105]. Researchers report the inhibitory function of *Fusarium oxysporum* fungus development by *B. subtilis* GM5 due to the presence of surfactin and fengycin at spore germination, sprouting tube formation, and hyphae branching phases [86]. Different studies associate an increase in antifungal activity with synergistic effects of fengycin and mycosubtilin [106]. The antibiotic capacity of LPs is associated with their ability to form micelles in the aqueous media and the membranotropic properties of these amphiphilic molecules, which disrupt the normal functioning of membranes due to their integration into the lipid bilayer and formation in pore membranes [103,107]. However, other evidence indicates that these molecules also activate the expression of some protective genes at very low concentrations. Thus, fungicins in plant cell culture potentially induce the components of the phenylpropanoid pathway [108], while surfactins induce active generation of hydrogen peroxide [24] and a number of components of the oxylipin signaling defense system against pathogens [75,109]. Diverse studies have reported the importance of these main antibiotics as functional components against a wide range of pathogenic microorganisms, including bacteria, fungi, and oomycetes [15,96,110]. Recent studies confirm that *B. subtilis* ABS-S14, effective in bio-control of *Penicillium digitatum*-caused green mold in mandarin, produces all three CLPs. Research by Waewethongrak and co-workers [111] on the effectiveness of partially purified compounds revealed that iturin A and fengycin inhibited the growth of *P. digitatum*, while surfactins did not exhibit a direct effect. *B. subtilis* FZB24 producing iturin-like LPs were efficacious against various phytopathogenic fungi [112]. Previous studies have revealed that *B. subtilis*, *B. atrophaeus*, and *B. amyloliquefaciens* producing iturin, fengycin, and surfactin possess high potential in the biotechnological industries due to their dynamic feature [95,113,114,115]. In addition, *Colletotrichum acutatum*-induced anthracnose symptoms in tamarillo fruits were completely inhibited by iturin A and fengycin C, and reduced by 76% when treated with *B. subtilis* EA-CB0015 [7]. Likewise, *B. subtilis* inhibits the development of peach brown rot by producing iturin [116]. Nevertheless, the contrast between *B. subtilis* strains was also observed in both individual activity and in the spectrum of LP level. Iturin, surfactin, and related compounds have been well known as the main components of the *B. subtilis* lipopeptide complex, whereas *P. chimensis* contains numerous homologs of bacillomycin and fenghin/plipastatin [114]. Fan et al. [49] suggested that gene *ppsB* is responsible for biosynthesis of fengycin, which represents the major antifungal compound produced by *B. subtilis* 9407 against *Botryosphaeria dothidea*. Biocontrol assays showed up to 50% reduction in *ppsB* gene expression compared with the wild-type strain, indicating the major role of fengycin in controlling apple ring rot disease caused by *B. dothidea* [49]. In addition, other antimicrobial peptide compounds such as bacilysin and subtilin, produced by *Bacillus* spp., *B. amyloliquefaciens*, and *B. subtilis* in particular, are also very efficient in obstructing different pathogens causing plant diseases [90,117,118,119,120,121].

Microbial antibiotics produced by *Bacillus* spp. may vary according to the species and strain [15]. It has been suggested that the combined biosynthesis of fengycin, bacillomycin, and iturin A by *B. subtilis* is related to the control of *Podosphaera fusca* in cucurbits [101]; iturin, bacilysin, and mersacidin produced by *B. subtilis* strain ME488 suppress the *Phytophthora blight* in pepper and *Fusarium wilt* in cucumber [122]. Mora et al. [97] investigated the presence of the antimicrobial peptide expressed by biosynthetic genes including *srfAA* (surfactin), *bacA* (bacylisin), *fenD* (fengycin), *ituC* (iturin), *spaS* (subtilin), and *bmyB* (bacyllomicin) in 184 isolates of *Bacillus* spp. obtained from plant environments (aerial, rhizosphere, and soil). According to different studies the ability of *Bacillus* spp. to suppress pathogens is associated with expression of the *bmyB*, *fenD*, *ituC*, *srfAA*, and *srfAB* genes [39,101,123,124]. Therefore *srfAA*, *bmyB*, *fenD*, and *bacA* have been introduced as the most frequent antimicrobial peptide gene markers [97].

Most *Bacillus* strains can produce varying amounts and types of LPs in response to the pathogens they encounter. [75]. For instance, *B. amyloliquefaciens* significantly increased iturin and fengycin biosynthesis in the presence of such pathogens as *Pythium aphanidermatum* and *F. oxysporum* [75]. The production of iturin and fengycin involved in the suppression of *Podosphaera fusca* by *B. subtilis* strains UMAF6614, UMAF6616, UMAF6639, and UMAF8561 and was demonstrated for cucurbits [101]. Toure et al. [39] reported that the strong protective effect of *B. subtilis* (strain GA1) on harvested apple fruit against grey mold caused by *B. cinerea* correlates with production of fengycins in infected apples. It was demonstrated that antibiotics gramicidin S and polymyxin B produced by *Brevibacillus brevis* and *Paenibacillus polymyxa*, respectively, controlled *Botrytis cinerea* (gray mold) of strawberry in both in vitro and in vivo [125]. *B. subtilis* (strain fmbJ) inhibited *Aspergillus flavus* development in corn by producing antibiotics like bacillomycin D with identical amino acid sequences (Asn–Tyr–Asn–Pro–Glu–Ser–Thr) [126]. Bull et al. [127] demonstrated that *Pseudomonas syringae* (strains ESC-10 and ESC-11) controlled *Penicillium digitatum* (green mold) of lemon by producing an antibiotic syringomycin E. However, the production of syringomycin E was never detected on the fruit/vegetables, casting doubt on the role of the antibiosis in controlling of the postharvest diseases and suggesting the operation of an alternative mechanism independent of syringomycin biosynthesis [127]. During Arabidopsis root colonization, *B. subtilis* (strain 6051) forms a stable and extensive biofilm and secretes surfactin component to protect plants against *Pseudomonas syringae* [128]. Asaka and Shoda [129] found that the surfactin and iturin A produced by *B. subtilis* (strain RB14) plays a pivotal role in the suppression of diseases caused by *Rhizoctonia solani* in tomato. Bacilopeptins, belonging to iturin-group antifungal antibiotics produced by *B. subtilis* (strain FR-2) isolated from rhizosphere of garlic plant infected by *Fusarium oxysporum* [130]. In addition, Yánez-Mendizábal et al. [131] demonstrated the ability of *B. subtilis* (strain CPA-8) in suppression of brown rot disease caused by *Monilinia* spp. in peaches governed by fengycin-like LPs. Likewise, CPA-8 also showed antifungal efficiency against other postharvest pathogens such as *M. laxa*, *Penicillium digitatum*, *P. italicum*, *Botrytis cinerea*, *M. fructicola*, and *P. expansum* [131]. A number of studies demonstrated that efficiency of *B. subtilis* in biocontrol of postharvest diseases is mainly attributable to the production of iturin, surfactin, and gramicidin [98,110,131]. Although large arrays of data indicate that antibiosis is an effective mechanism in controlling postharvest pathogens, some researchers have suggested that emphasis should be placed on the development of non-antibiotic producing microbial antagonists for the control of postharvest diseases as well [71,132]. However, it is obvious that LPs produced by different strains of bacteria play direct roles in the development of a host’s resistance to pathogens. Application of bacterial strains (especially endophytic) producing LPs have an advantage compared to the synthetic analogs due to their biodegradable nature, broad range of pH/temperature stability, and environmental safety, making this technology ideal for development in industrial areas including agriculture and food production [133].

Biosynthesis of antimicrobial compounds may be indirectly stimulated by siderophores—low molecular weight peptides that possess a high affinity for Fe ions. Antimicrobial compounds are released by various microorganisms to dissolve and assimilate Fe^3+^ that is inaccessible to biological systems [13]. Bacterial siderophores are classified into four groups: carboxylates, hydroxamates, phenolcatechelates, and pyoverdins. Competition for Fe^3+^ between PGPB and pathogenic microorganisms has been addressed as a main strategy for their function [88]. Pseudobacin is the most studied siderophore produced by *Pseudomonads* and possesses a strong antifungal effect. It was shown that pseudobacin produced by *P. putida* (strain WCS358), suppressed the development of *Ralstonia solanacearum* in eucalyptus, *Erwinia carotovora* in tobacco, and *Botrytis cinerea* in tomato [134]. Systemic resistance in plants will not emerge if bacteria lose the ability to synthesize the pseudobacin compounds [79]. Antibiotics and siderophores may additionally function as stress factors or signal inducing local and systemic resistance of host plant organism.

### 3.3. Synthesis of Hydrolytic Enzymes

Alternative possible mechanisms of antagonistic function of *Bacillus* bacteria can be attributed to the synthesis of extracellular hydrolases such as chitinases and β1,3-glucanases capable of destroying the structural polysaccharides of the fungal cell wall (chitin and glucans) and lysing the hyphae of fungi [85,98,131,132,133,134,135,136,137]. For a number of bacteria, a correlation between antagonistic activity to various pathogenic fungi and the synthesis of cellulases, mannanases, xylanases, proteases, and lipases has also been established [13,138]. A research on the complex of mycolytic enzymes of *B. subtilis* 739 showed that chitinase, chitosanase, β-1,3-glucanases, and proteases showed the most contribution to lysis of the native mycelium of various species of phytopathogenic fungi *Alternaria alternata*, *Bipolaris sorokiniana*, *Fusarium culmorum*, and *Rhizoctonia solani* [138]. Chitinolytic activity is often considered to be one of the most important criteria determining the antagonistic properties of bacteria. However, the question of the antifungal function of many bacterial chitinases remains controversial due to conflicting data associated with both the variety of bacterial strains that produce them and the structure of chitin of the cell walls of fungi that they affect [138]. In addition, when chitin is degraded by chitinases, chitooligosaccharides are initially formed, which effectively trigger pathogen-associated molecular patterns of ISR in plant organisms. The mechanism of their protective action includes activation of protective protein genes, including plant chitinases [139], enhancement of ROS generation, mainly H_2_O_2_, which in turn can also perform a signaling function by activating the genes regulating certain transcription factors or by interacting with other signaling components [140,141]. Notably, PGPB can induce the production of ROS in plant organisms, including H_2_O_2_ [142], which can be due to the induction of the systemic stability elements and biosynthesis of the oxidoreductases, i.e., oxalate oxidases by the bacteria [143,144].

The lytic enzymes synthesized by antagonistic *B. subtilis* NSRS 89-24 have proved to be very active in degrading the fungal cell walls of rice blast and sheath blight pathogens [20]. Swain et al. [145] investigated the interaction between *B. subtilis* and postharvest pathogen *F. oxysporum* of yam (*Dioscorea* spp.) tubers by scanning electron microscopy. Their results indicated that fungal cell wall lysis by *B. subtilis* was due to extracellular chitinase production [145]. It has been revealed that the strains of *B. subtilis* APEC170 and *Paenibacillus polymyxa* APEC136 diminish the symptoms of anthracnose caused by *Colletotrichum gloeosporioides* and *C. acutatum*, and white rot caused by *Botryosphaeria dothidea* in harvested apples by inhibition of mycelial growth of these pathogens. This can be attributed to the increased production of chitinase, amylase, and protease by the APEC170 strain, as well as elevated levels of amylase and protease in the APEC136 strain [36]. Kilani-Feki et al. [42] reported that chitosanases and proteases produced by *B. subtilis* V26 enhance harvested tomato resistance (up to 79%) to diseases caused by *Botrytis cinerea* during storage. Similarly, biocontrol efficiency of *B. subtilis* is mainly attributed to the production of antifungal compounds, including chitinases and glucanases [98,100,131]. Janisiewicz and Korsten [146] also reported that lytic enzymes produced by *Bacillus* spp. play a pivotal role in their biocontrol activity against *B. dothidea* that cause diseases in harvested fruit.

### 3.4. Induction of Systemic Resistance in Host Plant Organisms

*Bacillus* spp. suppress the development of different diseases in harvested fruits/vegetables not only directly through the synthesis of metabolites with fungicidal activity, but also indirectly, through the launch of multiple defense response mechanisms (Figure 1). These indirect mechanisms are linked to the formation of ISR and SAR (in whole host plant organisms) [147] and regulated by phytohormones such as SA, ABA, JA, ethylene [24,148], as well as CLPs [79]. To date, induction of auxins, cytokinins, gibberellins, ABA, JA, and SA has been detected in various bacteria [149,150,151,152]. The ability of PGPB to synthesize ABA, especially under stressful conditions, and to influence its level in plants was found in many strains of bacteria including the genera *Bacillus*, *Azospirillum*, *Pseudomonas*, *Brevibacterium*, and *Lysinibacillus* [149,153]. For example, inoculation of maize plants with ABA-producing *Azospirillum lipoferum* (strain USA59b) induced accumulation of ABA in plants and stimulated their growth under drought stress [154]. Using the “binary” system of ABA-deficient mutants of tomatoes *flacca* and *sitiens* and the rhizosphere bacterium *Bacillus megaterium* synthesizing ABA, it has been revealed that maintaining the normal level of ABA in tomato plants is extremely necessary for the growth of the host in both normal and stress conditions [80]. Different studies reported that phytopathogenic bacteria not only synthesize, but catabolize hormones and their precursors in plants [149]. Recently, bacterial strains have been isolated from the rhizosphere of rice with the capacity to dispose ABA with growth promotion in tomato plants through an ABA-dependent mechanism. Thus, under the influence of PGPB, an endogenous hormonal balance shift can occur in plants. It should be noted that the ability to produce phytohormones is found in both beneficial bacteria and pathogens [144]. In pathogens phytohormones are used for suppression of host defense systems while PGPB optimizes the hormonal balance of plants [144]. In addition, phytohormones produced by PGPB can, along with other compounds, launch certain mechanisms involved in protecting hosts against pathogens [13].

Some strains of *Bacillus* spp. produce low molecular weight VOCs (usually with a molecular weight of less than 300 kD), capable of easily propagating over long distances through diffusion in the air and pores in the soil. It has been established that exposure to VOCs can lead to a tissue-specific redistribution of endogenous auxins [155] and decrease in the ABA content in Arabidopsis [156]. In addition, these compounds are able to trigger ISR against pathogens and a number of abiotic stress factors [157]. *B. subtilis* UMAF6639 triggered in melon resistance to powdery mildew by activating JA- and SA-dependent protective reactions [158]. Some other bacteria are also able to induce defense responses in postharvest fruit by exploiting SA as a signaling molecule [159].

Almost all postharvest technologies manipulate the metabolism of the harvested product by inhibiting respiration rate of the product and the ethylene function as the key regulator of the ripening and senescence of fresh fruit and vegetables. Overproduction of ethylene leads to accelerated senescence and reduced shelf-life during postharvest storage [160,161]. To date, numerous findings have reported that PGPBs with capacity to produce the ACC-deaminase enzyme play a major role in modulating of ethylene levels in plants, thereby preventing harmful stress responses and promoting plant disease tolerance [13,162,163]. It was demonstrated in plants inoculated with ACC-deaminase-producing bacteria that ethylene production was significantly down-regulated, which ultimately prevented inhibition of plant growth induced by different stress factors (e.g., flooding/anoxia, drought, salinity, heavy metals, organic contaminants, the presence of fungal and bacterial pathogens, and nematodes). Since it is known that quick ripening is caused by ethylene, application of ACC-deaminase-producing bacteria during storage resulted in reduced ethylene levels in stored fruits/vegetables, which extended their aging process and in turn, their shelf life.

Different studies have suggested that the production of antifungal compounds by microbial antagonists in host cells also contributes to the induction of defense mechanisms and biologically control harvested fruit/vegetable diseases. *B. subtilis*, by producing iturin and fungicin, induces the expression of phenylpropanoid metabolism genes in plants, thereby triggering the mechanisms of ISR [103]. These data suggest that the pathway of SAR associated with ROS generation and triggered by SA, may also be involved in induction mechanisms of the protective system mediated by LPs. The strain *B. subtilis* 168 producing surfactin and fengycin enhanced the resistance of tomato and bean plants to *B. cinerea* by activating the enzymes of the lipoxygenase pathway [92], which drives synthesis of JA—an important molecule regulating ISR. Surfactin induced ISR in bean, melon, tomato, tobacco, and grapes, whereas fengycin induced protective responses in potatoes, tomatoes, and tobacco [164]. The *B. amyloliquefaciens* strain producing surfactin induced ISR in *Brassica napus* against *Botrytis cinerea* [165], as well as *Bacillus amyloliquefaciens* FZB42 induced ISR in lettuce against *Rhizoctonia solani*, by the JA/ethylene-dependent signaling protective pathway through induction of PDF 1.2 gene expression [166]. It has been found that mycosubtilin triggers protective reactions in grape plants [164]. However, recombinant strains of *B. amyloliquefaciens* FZB42 deficient in the synthesis of surfactin (CH1), as well as in the synthesis of surfactin, fengycin, and bacillomycin D (CH5) lost the ability to increase the resistance of lettuce to Rhizoctonia [166]. It has been shown that *B. subtilis* strain BBG111 induces ISR in *R. solani* in the rhizosphere of rice due to the secretion of fengycin and surfactin, thereby causing a hypersensitive reaction and cell death. The immune responses develop over the JA/ethylene-, ABA- and auxin-dependent defense signaling pathways, which block the growth and development of the pathogen in the early stages of pathogenesis [167]. Future research should provide additional insight into the subtle mechanisms by which LPs interact with plant cells. By using genomic shuffling, Zhang and co-workers [168] generated a *B. amyloliquefaciens* strain (FMB72) synthesizing 8.3 times more fengycin than the original strain of *B. amyloliquefaciens* ES-2-4 isolated from *Scutellaria baicalensis* Georgi. This strain possessed high biocidal activity against pathogens. Evidence from numerous studies indicate that *Bacillus* spp. elicit defense mechanisms in harvested fruits/vegetables and control of the postharvest decay through the accumulation of phytoalexins (scoparone and scopoletin) [169,170]. Complementary studies demonstrate that the application of the microbial product based on the *B. subtilis* (strain Ch-13) generate twice the protective response in potato tubers and increase the activity of peroxidase, ascorbic acid content, and the formation of phytoalexins in tubers during storage at 18 °C [55].

The ISR caused by endophytic bacteria is preserved in plants for a long time and effectively works against pathogens under storage conditions [59]. It manifests in early cascade and rapid accumulation of ROS, including H_2_O_2_, after the onset of infection, and corresponds with the up-regulation of redox-sensitive transcription factors and PR genes. Thus, *Pseudomonas putida* LSW17S induced a rapid accumulation of transcription PR genes and production of H_2_O_2_ in tomato plants infected by *P. syringae* pv. tomato DC3000, which inhibited pathogen development [171]. The reduced development of the fungus *Rhizopus stolonifer* on peach fruit treated with *B. cereus* AR156 and *B. subtilis* SM21 was associated with the generation of H_2_O_2_, and overexpression of chitinase genes, β1,3-glucanase, and phenylalanine–ammonium–lyase and the activity of their protein products [53]. *B. subtilis* BSCBE4 and *P. chlororaphis* PA23 activated peroxidases and polyphenol oxidases catalyzing the final lignin biosynthesis reactions in pepper seedlings infected with *Pythium aphanidermatum* [172]. *B. subtilis* (strain Bs16), *Trichoderma viride* (strains Tv1 and Tv13), *Pseudomonas fluorescens* (strains Pf1 and Py15) activate production of peroxidase, polyphenol oxidase, phenylalanine–ammonia–lyase and protect host plant tissues against various pathogens [173]. During development of ISR induced by endophytic bacteria, generation of ROS in plants can play a critical role in the formation of the priming effect. The priming phenomenon in the host genome under the influence of bacterial agents causes hypersensitivity to the effects of foreign substances, is characterized by faster and stronger activation of cellular mechanisms of plant protection under pathogens or insect invasion, and can last for a rather long time. This will lead to an increase of plant resistance. It is suggested that such priming in response to bacterial infection is associated with a change in the status of DNA methylation in the plant genome [174]. Nevertheless, despite the number of findings of the different *Bacillus*-induced defense responses in harvested fruit/vegetables against pathogens, the undelaying protective mechanisms still remain unclear and require more investigation.

## 4. Methods of Application

**Figure 2:**
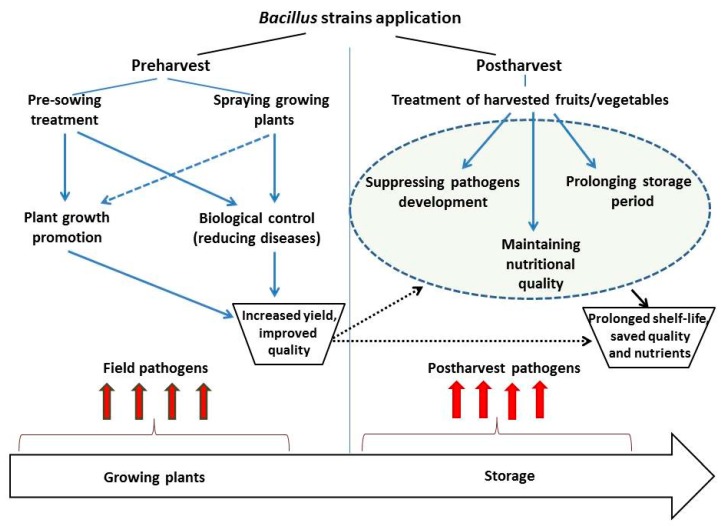
Scheme of *Bacillus* strains application strategies for diseases management of harvested fruits/vegetables during storage.

The effectiveness of potential microbial antagonists in suppressing pathogens in harvested fruits/vegetables depends on both characteristics of selected strain and application method. In general, microbial agents can be applied using pre- or post-harvest strategies (Figure 2).

### 4.1. Preharvest Application

Pathogens often infect fruit/vegetables in the field and live in plant tissues during vegetation season without causing any symptoms; however, these “hidden” infections may begin to develop during storage and become the major decay factor leading to significant food losses [175]. Previous research suggests that the application of microbial inoculants, particularly *B. subtilis*, reduces stress induced defects and positively affects crop yield during storage [16,78]. It has been shown that pre-sowing treatment of potato (*Solanum tuberosum* L.) tubers with endophytic *B. subtilis* strains 10-4 and 26D, positively affects plant growth, development, and yield of potato in the field, results in decreased infestation of tubers by pathogenic micromycetes *Aspergillus*, *Pennicillium*, and *Alternaria* and completely suppresses the development of *Cladosporium*, *Fusarium*, and *Mucor*, compared to non-inoculated control tubers both after harvesting and during storage [60]. This implies that preharvest application(s) of microbial agents are often effective in controlling postharvest diseases of fruits/vegetables [72,176,177,178]. Preharvest application of *B. subtilis* under field conditions results in colonization of the apple fruit surface by the microbial antagonist, which effectively controls the postharvest pathogens *Penicillium expansum* and *Botrytis cinerea* of apples [179]. In some cases infection occurs shortly before harvesting. The symptoms of these infections may not be apparent during harvest and become visible in the postharvest period especially when the proper conditions are available for pathogen development. It has been reported that late-developing infections are often caused by pathogens such as *B. cinerea*, *Monilinia fructicola*, *Sclerotium rolfsii*, and *Geotrichum candidum* [175,180]. According to Ippolito and Nigro [176] application of antagonistic microorganisms immediately before harvest contributes to the colonization of the fruit surfaces and protects them from pathogens during storage. Although this approach has been successful in certain cases, it has generally not become commercially viable because of the poor survival rate of microbial antagonists under field conditions. Nevertheless, there is evidence for the effectiveness of *Bacillus* spp. in the bio-control of postharvest avocado diseases, by use of the bacteria during vegetation, and in the pre-vegetation period before the laying of fruit for storage [54,181].

### 4.2. Postharvest Application

Postharvest application of microbial antagonists has been introduced as a proper and practical method for control of harvested fruit and vegetables disease. According to this method, preparations containing microbial antagonists can be applied as postharvest sprays or as solutions in harvested fruit/vegetables (Figure 2) [178,182].

However, the application of one individual microbial antagonist cannot completely prevent all postharvest fruits/vegetables decay during storage. It is difficult to select an individual effective microbial strain with a broad spectrum of activity against wide range of pathogens [74,132,182,183]. Some manufacturers use mixtures of bacterial strains to enhance the protective properties of biological preparations. For example, ‘‘Companion’’ (Growth Products Ltd., USA) is formulated from a mix of *Bacillus* spp. (*B. subtilis* GB03, *B. lichenoformis*, and *B. megaterium*) and ‘‘Bactril’’ (Biopharmatec, Russia) contains *B. nigrum* 132, *B. subtilis* MBI600, and *Bradyrhizobium japonicum* [57]. *Bacillus* spp. also can be considered one element, applied alongside other biological and physical methods, as part of an integrated vision of disease management. For example, mixtures of diverse antagonistic microorganisms with a broad spectrum of microbial activity and combination of different bio-controlling traits, leads to the control of two or more post-harvest diseases when applied under different environmental conditions [137]. The effectiveness of microbial antagonists in controlling postharvest decay on fruits/vegetables also can be enhanced by the addition of enhancer effectors, such as calcium chloride, calcium propionate, sodium bicarbonate, ammonium molybdate, sodium carbonate, potassium metabisulphite, SA, etc. Likewise, microbial antagonists can be combined with wax agents in post and preharvest periods [183,184]. Integration of microbial antagonists with physical methods, such as curing or heat treatments, could enhance the bio-efficacy of microbial agents as well [74,137]. Given the huge body of available knowledge about the mechanisms underlying the protective function of microbial antagonists (i.e., *Bacillus* spp.), biologically active compounds, and induced resistance, the introduction of more effective formulations, application methods and combinations with additional approaches for additive and/or synergistic effects, will not be unlikely [183].

## 5. Development and Commercialization of *Bacillus*-Based Postharvest Biocontrol Products

The first microorganism patented as a postharvest bio-controlling agent effective against brown rot of stone fruits was *B. subtilis* strain B-3 (USA) [45]. A pilot experiment using *B. subtilis* B-3 under commercial conditions for postharvest control of peach brown rot caused by *Monilinia fructicola* was conducted by Pusey et al. [184], where the biological agent was effectively incorporated into wax commonly used in three different sites: packing lines in Byron (Georgia), Clemson (South Carolina), and a commercial packing house in Musella (Georgia). In packing line tests, the number of colony forming units of the bacterium *B. subtilis* added to each fruit was between 2 × 10^7^ and 7 × 10^7^ for B-3 from flask cultures, and 2 × 10^9^ for B-3 from the fermenter culture. In a test at Byron, *B. subtilis* B-3 cultures, applied fresh or as a stored paste/powder, were equally effective as the chemical fungicide benomyl in control of brown rot. In a similar test at Clemson, stored forms of B-3 from both flask and fermenter cultures were as effective as benomyl [184]. In recent years, significant attention has been placed on postharvest bio-controlling agents formulated from antagonistic microorganisms, including *Bacillus* spp. To date, several commercial microbial antagonists of the *Bacillus* genus, particularly *B. subtilis*, have been reported to control postharvest diseases in fruits/vegetables under laboratory and field conditions (Table 2). For example, a new biofungicide based on the *B. subtilis* strain QST 713, registered in the USA (Serenade, AgraQuest), has been formulated to control fungi of the *Fusarium*, *Pythium*, and *Phytophthora* genera on fruit and vegetables during storage [185]. Biocontrol agents containing *B. subtilis* strains FZB24 and QST713 (Serenade and Rhio-plus) have already been commercially applied to control brown rot, powdery mildew, root rots, fire blight, and late blight on fruit/vegetables [137,186]. It has been shown that the commercial product based on *B. subtilis* strain QST 713 (Rhapsody) significantly reduced the development of postharvest rot in tomato [66].

Different research carried out under laboratory conditions demonstrated that *Bacillus* spp. strains have a notable potential for being developed into a postharvest bio-controlling agent, which can be applied to different fruits/vegetables against a wide range of pathogens (Table 1). For example, *Bacillus* spp. C06 suppresses *Monilinia fructicola* (brown rot) incidence by 92% and reduced lesion diameter by 88% [67]. *Bacillus* spp. T03-c reduced incidence and lesion diameter by 40% and 62%, respectively. Treatments with strains of MA-4, T03-c, and C06 significantly controlled brown rot by 91%, 100%, and 100%, respectively [67]. Two bio-control products based on *B. amyloliquefaciens* CPA8 have been developed as effective agents to control postharvest brown rot diseases in stone fruits. Gotor-Vila et al. [69] demonstrated that these products reduce the development of diseases caused by *Monilinia* spp. (more than 44.4%) in peaches, cherries, nectarines, plums, and apricots. However, one of the main factors restricting development and commercialization of postharvest bio-controlling products based on *Bacillus* spp. is the lack of knowledge about their multiple modes of actions in *Bacillus* spp.–hosts–pathogen interactions. Furthermore, success in the commercialization of prospective *Bacillus* spp. strains depends on the links between scientific organizations and industries as well. It follows that, even if microbial antagonists represent all the desirable characteristics, the commercial success requires economic and viable market demand.

## 6. Conclusions and Future Prospects

*Bacillus* spp. positively affect the postharvest physiology of fruits/vegetables by enhancing their resistance against different postharvest pathogens resulting in a prolonged storage period and extended “marketing” life, and preserving freshness and nutritional quality. *Bacillus* strains (especially endophytic) may be developed as effective bio-control agents to decrease postharvest decay of fruits/vegetables. Developing these microbial antagonists as commercial agents for postharvest biological control is a strong prospect. Despite *Bacillus* spp. having a clear advantage as an eco-friendly strategy for preventing food losses during storage, the knowledge about the mode of action on postharvest physiology and preservation under pathogenic infection is limited and requires detailed investigation Therefore, understanding the mechanism/s of function for these bacteria will play an important role in the promotion of *Bacillus*-based products to support green technology in the agricultural and food industries.

## Figures and Tables

**Table 1:** Examples of successful *Bacillus* spp. application for control of postharvest diseases of fruits/vegetables.

Bacterial Inoculant	Disease/Pathogen	Fruits/ Vegetables	Reference(s)
*B. subtilis*	Brown rot (*Lasiodiplodia theobromae*)	Apricot	[45]
	Stem and rot (*Botryodiplodia theobromae Pat*.)	Avocado	[64]
	Botrytis rot (*Botrytis cinerea*)	Cherry	[52]
	Green mold (*Penicillium digitatum*)	Citrus	[37]
	Sour rot (*Geotrichum candidum Link*)	Citrus	[37]
	Stem and rot (*Botryodiplodia theobromae, Phomopsis citri H.S. Fawc*., *Alternaria citri Ell.*, and *Pierce*)	Citrus	[37]
	Alternaria rot (*A. alternata (Fr.) Keissler*)	Litchi	[33]
	Brown rot (*Lasiodiplodia theobromae*)	Nectarine	[45]
	Brown rot (*L. theobromae*)	Peach	[45]
	Brown rot (*L. theobromae*)	Plum	[45]
	Fungal rot (*Aspergillus niger*, *Botryodiploidia theobromae*, *Penicillium oxalicum*)	Yam	[50]
	Gray mold (*Botrytis cinerea*)	Strawberry	[40]
	Alternaria rot (*Alternaria alternata*)	Muskmelon	[65]
	*A. alternata*	Melon	[34]
	*Aspergillus*, *Pennicillium*, *Alternaria*, *Cladosporium*, *Fusarium*, *Mucor*	Potato	[60]
	*Colletotrichum acutatum*	Apple	[35]
	*Botrytis cinerea*	Apple	[39]
	*Botryosphaeria dothidea*, *Diaporthe actinidiae*, *Botrytis cinerea*	Kiwifruit	[35]
	Anthracnose (*Colletotrichum gloeosporioides*, *C. acutatum*), white rot (*B. dothidea*)	Apple	[36]
	Ring rot (*B. dothidea*)	Apple	[49]
	Rot (*Penicillium* sp., *Rhizopus stolonifer*)	Tomato	[66]
	Grey mold (*Botrytis cinerea)*	Tomato	[42]
	Brown rot (*Monilinia fructicola*)	Peach	[67]
	Brown rot (*Monilinia* spp.)	Peach	[68]
	Rot (*Rhizopus*)	Peach	[53]
	Green mold (*Penicillium digitatum*)	Citrus	[46]
*B. amyloliquefaciens*	Brown rot (*Monilinia* spp.)	Peaches	[69]
		Nectarines	
		Flat peaches	
		Cherries	
		Apricots	
		Plums	
*B. licheniformis (Weigmann) Verhoeven*	Anthracnose (*Colletotrichum gloeosporioides*) and stem end rot (*Dothiorella gregaria* Sacc.)	Mango	[70]
*B. licheniformis*	Grey mold (*Botrytis mali*)	Apple	[47]
*B. pumilus*	Gray mold (*B. cinerea*)	Pear	[38]

**Table 2:** Biocontrol products based on *Bacillus* spp. developed and commercialized to control postharvest diseases of fruits/vegetables.

Product	Microbial Agent	Fruits/Vegetables	Target Disease(s)	Manufacturer/Distributor
Rhio-plus	*B. subtilis* FZB 24	Potatoes and other vegetables	Powdery mildew and root rots	KFZB Biotechnick, Germany
Serenade	*B. subtilis QST713*	Apple, pear, grapes, tomato, potato	Powdery mildew, late blight, brown rot, and fire blight	AgraQuest. Inc., USA
Phytosporin-M Golden Authum, AntiGnil Phytosporin M	*B. subtilis 26D*	Carrot, tomato, cabbage, sugar beet, potato	Rots, mold	Bashinkom, Russia
Rhapsody^®^	*B. subtilis* QST 713	Tomato	Rots	Bayer, Canada

## References

[1] FAO (2015). Food Losses and Waste. http://www.fao.org/food-loss-and-food-waste/en/.

[2] Hodges R.J., Buzby J.C., Bennett B. (2010). Postharvest losses and waste in developed and less developed countries: Opportunities to improve resource use. J. Agric. Sci..

[3] Buchholz F., Kostic T., Sessitsch A., Mitter B. (2018). The potential of plant microbiota in reducing postharvest food loss. Microb. Biotechnol..

[4] Droby S., Wisniewski M., Teixidó N., Spadaro D., Jijakli M.H. (2016). The science, development, and commercialization of postharvest biocontrol products. Postharvest Biol. Technol..

[5] Wisniewski M., Droby S., John N., Liu J., Schena L. (2016). Alternative management technologies for postharvest disease control: The journey from simplicity to complexity. Postharvest Biol. Technol..

[6] Dimkpa C.O., Merten D., Svatos A., Büchel G., Kothe E. (2009). Siderophores mediate reduced and increased uptake of cadmium by *Streptomyces tendae* F4 and sunflower (*Helianthus annuus*), respectively. J. Appl. Microbiol..

[7] Arroyave-Toroa J.J., Mosquera S., Villegas-Escobar V. (2017). Biocontrol activity of *Bacillus subtilis* EA-CB0015 cells and lipopeptides against postharvest fungal pathogens. Biol. Control.

[8] Sarma B.K., Yadav K.S., Singh D.P., Singh H.B. (2012). Rhizobacteria mediated induced systemic tolerance in plants: Prospects for abiotic stress management. Bacteria in Agrobiology: Stress Management.

[9] Van Loon L.C. (2007). Plant responses to plant growth-promoting rhizobacteria. Eur. J. Plant Pathol..

[10] Baez-Rogelio A., Morales-García Y.E., Quintero-Hernández V., Muñoz-Rojas J. (2016). Next generation of microbial inoculants for agriculture and bioremediation. Microb. Biotechnol..

[11] Lastochkina O., Pusenkova L., Yuldashev R., Babaev M., Garipova S., Blagova D., Khairullin R., Aliniaeifard S. (2017). Effects of *Bacillus subtilis* on some physiological and biochemical parameters of *Triticum aestivum* L. (wheat) under salinity. Plant Physiol. Biochem..

[12] Seifikalhor M.S., Aliniaeifard S., Self M., Javadi E., Bernard F., Li T., Lastochkina O. (2018). Rhisobacteria *Bacillus subtilis* reduces toxic effects of high electrical conductivity in soilless culture of lettuce. Acta Hortic..

[13] Maksimov I.V., Veselova S.V., Nuzhnaya T.V., Sarvarova E.R., Khairullin R.M. (2015). Plant growth promoting bacteria in regulation of plant resistance to stress factors. Rus. J. Plant Physiol..

[14] Nagórska K., Bikowski M., Obuchowski M. (2007). Multicellular behaviour and production of a wide variety of toxic substances support usage of *Bacillus subtilis* as a powerful biocontrol agent. Acta Biochim. Pol..

[15] Ongena M., Jacques P. (2008). *Bacillus* lipopeptides: Versatile weapons for plant disease biocontrol. Trends Microbiol..

[16] Gao H., Xu X., Dai Y., He H. (2016). Isolation, identification and characterization of *Bacillus subtilis* CF-3, a bacterium from fermented bean curd for controlling postharvest diseases of peach fruit. Food Sci. Technol. Res..

[17] Knox O.G.G., Killham K., Leifert C. (2000). Effects of increased nitrate availability on the control of plant pathogenic fungi by the soil bacterium *Bacillus subtilis*. Appl. Soil Ecol..

[18] Mannanov R.N., Sattarova R.K. (2001). Antibiotics produced by *Bacillus bacteria*. Chem. Nat. Compd..

[19] Jiang Y.M., Zhu X.R., Li Y.B. (2001). Postharvest control of litchi fruit rot by *Bacillus subtilis*. Food Sci. Technol..

[20] Leelasuphakul W., Sivanunsakul P., Phongpaichit S. (2006). Purification, characterization and synergistic activity of b1,3-glucanase and antibiotic extract from an antagonistic *Bacillus subtilis* NSRS 89-24 against rice blast and sheath blight pathogens. Enzyme Microb. Technol..

[21] Aouadhi C., Rouissi Z., Kmiha S., Mejri S., Maaroufi A. (2016). Effect of sporulation conditions on the resistance of *Bacillus sporothermodurans* spores to nisin and heat. Food Microbiol..

[22] Gupta V., Bochow H., Dolej S., Dolej S., Fischer I. (2000). Plant growth-promoting *Bacillus subtilis* strain as potential inducer of systemic resistance in tomato against *Fusarium* wilt. J. Plant Dis. Protect..

[23] Beneduzi A., Ambrosini A., Passaglia L. (2012). Plant growth-promoting rhizobacteria (PGPR): Their potential as antagonists and biocontrol agents. Genet. Mol. Biol..

[24] García-Gutiérrez L., Zeriouh H., Romero D., Cubero J., Vicente A., Pérez-García A. (2013). The antagonistic strain *Bacillus subtilis* UMAF6639 also confers protection to melon plants against cucurbit powdery mildew by activation of jasmonate—And salicylic acid-dependent defense responses. Microb. Biotechnol..

[25] Pusenkova L.I., Il’yasova E.Y., Lastochkina O.V., Maksimov I.V., Leonova S.A. (2016). Changes in the species composition of the rhizosphereand phyllosphere of sugar beet under the impact of biological preparations based on endophytic bacteria and their metabolites. Eurasian Soil Sci..

[26] Egamberdieva D., Wirth S.J., Shurigin V.V., Hashem A., Abd Allah E.F. (2017). Endophytic bacteria improve plant growth, symbiotic performance of chickpea (*Cicer arietinum* L.) and induce suppression of root rot caused by *Fusarium solani* under salt stress. Front. Microbiol..

[27] Shafi O., Tian H., Ji M. (2017). *Bacillus* species as versatile weapons for plant pathogens: A review. Biotechnol. Biotechnol. Equip..

[28] Bochow H., El-Sayed S.F., Junge H., Stavropoulou A., Schmiedeknecht G. (2001). Use of *Bacillus subtilis* as biocontrol agent. IV. Salt-stress tolerance induction by *Bacillus subtilis* FZB24 seed treatment in tropical vegetable field crops, and its mode of action. Zeitschrift fur Pflanzenkrankheiten und Pflanzenschutz..

[29] Saleh S.A., Heuberger H., Schnitzler W.H. (2005). Alleviation of salinity effect on artichoke productivity by *Bacillus subtilis* FZB24, supplemental Ca and micronutrients. J. Appl. Bot. Food Qual..

[30] Turan M., Ekinci M., Yıldırım E., Güneş K., Karagöz K., Kotan R., Dursun A. (2014). Plant growth-promoting rhizobacteria improved growth, nutrient, and hormone content in cabbage (*Brassica oleracea*) seedlings. Turk. J. Agric. For..

[31] Berg G. (2009). Plant-microbe interactions promoting plant growth and health: Perspectives for controlled use of microorganisms in agriculture. Appl. Microbiol. Biotechnol..

[32] Pandey P.K., Singh M.C., Singh S.S., Kumar A.K., Pathak M.M., Shakywar R.C., Pandey A.K. (2017). Inside the plants: Endophytic bacteria and their functional attributes for plant growth promotion. Int. J. Curr. Microbiol. Appl. Sci..

[33] Jiang Y.M., Chen F., Li Y.B., Liu S.X. (2001). A preliminary study on the biological control of postharvest diseases of Litchi fruit. J. Fruit Sci..

[34] Wang Y., Xu Z., Zhu P., Liu P., Zhang Z., Mastuda Y., Toyoda H., Xu L. (2010). Postharvest biological control of melon pathogens using *Bacillus subtilis* EXWB1. J. Plant Pathol..

[35] Kim G.H., Koh Y.J., Jung J.S., Hur J.S. (2015). Control of postharvest fruit rot diseases of kiwifruit by antagonistic bacterium *Bacillus subtilis*. Acta Hortic..

[36] Kim Y.S., Balaraju K., Jeon Y. (2016). Effects of rhizobacteria *Paenibacillus polymyxa* APEC136 and *Bacillus subtilis* APEC170 on biocontrol of postharvest pathogens of apple fruits. J. Zhejiang Univ. Sci. B.

[37] Singh V., Deverall B.J. (1984). *Bacillus subtilis* as a control agent against fungal pathogens of citrus fruit. Trans. Br. Mycol. Soc..

[38] Mari M., Guizzardi M., Pratella G.C. (1996). Biological control of gray mold in pears by antagonistic bacteria. Biol. Control.

[39] Touré Y., Ongena M., Jacques P., Guiro A., Thonart P. (2004). Role of lipopeptides produced by *Bacillus subtilis* GA1 in the reduction of grey mould disease caused by *Botrytis cinerea* on apple. J. Appl. Microbiol..

[40] Zhao Y., Shao X.F., Tu K., Chen J.K. (2007). Inhibitory effect of *Bacillus subtilis* B10 on the diseases of postharvest strawberry. J. Fruit Sci..

[41] Jamalizadeh M., Etebarian H.R., Aminian H., Alizadeh A. (2010). Biological control of *Botrytis mali* on apple fruit by use of *Bacillus* bacteria, isolated from the rhizosphere of wheat. Arch. Phytopathol. Plant Protect..

[42] Kilani-Feki O., Ben Khedher S., Dammak M., Kamoun A., Jabnoun-Khiareddine H., Daami-Remadi M., Touns S. (2016). Improvement of antifungal metabolites production by *Bacillus subtilis* V26 for biocontrol of tomato postharvest disease. Biol. Control.

[43] Miller A.R. (2003). Harvest and Handling Injury: Physiology, Biochemistry, and Detection. Postharvest Physiology and Pathology of Vegetables.

[44] Tronsmo A., Denis C. (1977). The use of *Trichoderma* species to control strawberry fruit rots. Neth. J. Plant Pathol..

[45] Pusey P.L., Wilson C.L. (1984). Postharvest biological control of stone fruit brown rot by *Bacillus subtilis*. Plant Dis..

[46] Mohammadi P., Tozlu E., Kotan R., Şenol Kotan M. (2017). Potential of some bacteria for biological control of postharvest citrus green mould caused by *Penicillium digitatum*. Plant Protect. Sci..

[47] Jamalizadeh M., Etebarian H.R., Alizadeh A.A., Aminian H. (2008). Biological control of gray mold on apple fruits by *Bacillus licheniformis* (EN74-1). Phytoparasitica.

[48] Alfonzo A., Conigliaro G., Torta L., Burruano S., Moschetti G. (2009). Antagonism of *Bacillus subtilis* strain AG1 against vine wood fungal pathogens. Phytopathol. Mediterr..

[49] Fan H., Ru J., Zhang Y., Wang Q., Li Y. (2017). Fengycin produced by *Bacillus subtilis* 9407 plays a major role in the biocontrol of apple ring rot disease. Microbiol. Res..

[50] Okigbo R.N. (2005). Biological control of postharvest fungal rot of yam (*Dioscorea* spp.) with *Bacillus subtilis*. Mycopathologia.

[51] Qi D., Hui M., Liang Q., Niu T. (2005). Postharvest biological control of blue mold and black spot on apple-pear (Pyrus bretschneideri Rehd.) fruit by *Bacillus subtilis* H110. Chin. J. Appl. Environ. Biol..

[52] Utkhede R.S., Sholberg P.L. (1986). In vitro inhibition of plant pathogens: *Bacillus subtilis* and *Enterobacter aerogenes* in vivo control of two postharvest cherry diseases. Can. J. Microbiol..

[53] Wang X., Wang J., Jin P., Zheng Y. (2013). Investigating the efficacy of *Bacillus subtilis* SM21 on controlling *Rhizopus* rot in peach fruit. Int. J. Food Microbiol..

[54] Korsten L., De Villiers E.E., De Jager E.S., Cook N., Kotzé J.M. (1991). Biological control of avocado postharvest diseases. South African Avocado Growers’ Association Yearbook.

[55] Chebotar V.K., Kiprushkina E.I. (2015). Application of microbial preparations in potato storage technologies. Dostizheniya Nauki i Tekhniki APK.

[56] Kavino M., Manoranjitham S.K., Vijayakumar N.K.R. (2016). Plant growth stimulation and biocontrol of Fusarium wilt (*Fusarium oxysporium* f. sp. cubene) by coinoculation of banana (Musa sp.) plantlets with PGPR and endophytes. Recent Trends in PGPR Research for Sustainable Crop Productivity, Proceedings of the 4th Asian PGPR Conference, Hanoi, Vietnam, 3–6 May 2016.

[57] Maksimov I.V., Khairullin R.M., Gupta V.K., Sharma G.D., Tuohy M.G., Gaur R. (2016). The role of *Bacillus* bacterium in formation of plant defense: Mechanism and reaction. The Handbook of Microbial Bioresourses.

[58] Rahman S., Rahman L., Khalil A.T., Ali N., Zia D., Ali M., Shinwari Z.K. (2019). Endophyte-mediated synthesis of silver nanoparticles and their biological applications. Appl. Microbiol. Biotechnol..

[59] Maksimov I.V., Pusenkova L.I., Abizgildina R.R. (2011). Biopreparation with endophytic bacterium Bacillus subtilis 26D created postharvest protecting effect in potato tubers. Agrochemistry.

[60] Lastochkina O.V., Yuldashev R.A., Pusenkova L.I. (2015). Assessment of the influence of Bacillus subtilis bacterial strains in mix with salicylic acid on productivity and infection of potato tubers. Agric. Sci. Innov. Dev. AIC.

[61] Aghdam M.S., Asghari M., Babalar M., Sarcheshmeh M.A.A. (2016). Impact of salicylic acid on postharvest physiology of fruits and vegetables. Eco-Friendly Technology for Postharvest Produce Quality.

[62] Fung R., Wang C., Smith D., Gross K., Tian M. (2004). MeSA and MeJA increase steady-state transcript levels of alternative oxidase and resistance against chilling injury in sweet peppers (*Capsicum annuum* L.). Plant Sci..

[63] Cai C., Xu C.J., Li X., Ferguson I., Chen K.S. (2006). Accumulation of lignin in relation to change in activities of lignification enzymes in loquat fruit flesh after harvest. Postharvest Biol. Technol..

[64] Demoz B.T., Korsten L. (2006). *Bacillus subtilis* attachment, colonization, and survival on avocado flowers and its mode of action on stem-end rot pathogens. Biol. Control.

[65] Yang D.M., Bi Y., Chen X.R., Ge Y.H., Zhao J. (2006). Biological control of postharvest diseases with *Bacillus subtilis* (B1 strain) on muskmelons (*Cucumis melo* L. cv. Yindi). Acta Hortic..

[66] Punjia Z.K., Rodriguez G., Tirajoh A. (2016). Effects of *Bacillus subtilis* strain QST 713 and storage temperatures on post-harvest disease development on greenhouse tomatoes. Crop Prot..

[67] Zhou T., Schneider K.E., Li X. (2008). Development of biocontrol agents from food microbial isolates for controlling post-harvest peach brown rot caused by *Monilinia fructicola*. Int. J. Food Microbiol..

[68] Yánez-Mendizábal V., Zeriouh H., Viñas I., Torres R., Usall J., de Vicente A., Pérez-García A., Teixidó N. (2012). Biological control of peach brown rot (*Monilinia* spp.) by *Bacillus subtilis* CPA-8 is based on production of fengycin-like lipopeptides. Eur. J. Plant Pathol..

[69] Gotor-Vila A., Usall J., Torres R., Solsona C., Teixidó N. (2017). Biocontrol products based on *Bacillus amyloliquefaciens* CPA-8 using fluid-bed spray-drying process to control postharvest brown rot in stone fruit. LWT Food Sci. Technol..

[70] Govender V., Korsten L., Sivakumar D. (2005). Semi-commercial evaluation of *Bacillus licheniformis* to control mango postharvest diseases in South Africa. Postharvest Biol. Technol..

[71] Droby S., Chalutz E., Wilson C.L., Wisniewski M.E. (1992). Biological control of postharvest diseases: A promising alternative to the use of synthetic fungicides. Phytoparasitica.

[72] Janisiewicz W.J., Tworkoski T.J., Sharer C. (2000). Characterizing the mechanism of biological control of postharvest diseases on fruit with a simple method to study competition for nutrients. Phytopathology.

[73] Jijakli M.H., Grevesse C., Lepoivre P. (2001). Modes of action of biocontrol agents of postharvest diseases: Challenges and difficulties. Bulletin-OILB/SROP.

[74] El-Ghaouth A., Wilson C.L., Wisniewski M.E., Samh N. (2004). Biologically based alternatives to synthetic fungicides for the postharvest diseases of fruit and vegetables. Diseases of Fruit and Vegetables.

[75] Cawoy H., Debois D., Franzil L., De Pauw E., Thonart P., Ongena M. (2015). Lipopeptides as main ingredients for inhibition of fungal phytopathogens by *Bacillus subtilis*/*amyloliquefaciens*. Microb. Biotechnol..

[76] Baindara P., Korpole S. (2016). Lipopeptides: Status and strategies to control fungal infection. Recent Trends in Antifungal Agents and Antifungal Therapy.

[77] Van der Ent S.S., Van Wees S.C.M., Pieterse C.M.J. (2009). Jasmonate signaling in plant interactions with resistance-inducing beneficial microbes. Phytochemistry.

[78] Maksimov I.V., Abizgildina R.R., Pusenkova L.I. (2011). Plant growth promoting rhizobacteria as alternative to chemical crop protectors from pathogens (Review). Appl. Biochem. Microbiol..

[79] De Vleesschauwer D., Höfte M. (2009). Rhizobacteria-Induced Systemic Resistance. Adv. Bot. Res..

[80] Bacon W.C., Yates E.I., Hinton M.D., Meredith F. (2001). Biological control of *Fusarium moniliforme* in maize. Environ. Health Perspect..

[81] Porcel R., Zamarreño A.M., García-Mina J.M., Aroca R. (2014). Involvement of plant endogenous ABA in *Bacillus megaterium* PGPR activity in tomato plants. BMC Plant Biol..

[82] Wilson C.L., Wisniewski M.E. (1989). Biological control of postharvest diseases of fruit and vegetables: An emerging technology. Annu. Rev. Phytopathol..

[83] Droby S., Chalutz E., Wilson C.L., Wisniewski M.E. (1994). Mode of action of biological agents of postharvest diseases. Biological Control of Postharvest Diseases—Theory and Practice.

[84] Droby S., Chalutz E., Wilson C.L., Wisniewski M. (1989). Characterization of the biocontrol activity of *Debaryomyces hansenii* in the control of *Penicillium digitatum* on grapefruit. Can. J. Microbiol..

[85] Castoria R., de Curtis F., Lima G., Caputo L., Pacifico S., de Cicco V. (2001). *Aureobasidium pullulans* (LS-30), an antagonist of postharvest pathogens of fruits: Study on its mode of action. Postharvest Biol. Technol..

[86] Mardanova A.M., Hadieva G.F., Lutfullin M.T., Khilyas I.V., Minnullina L.F., Gilyazeva A.G., Bogomolnaya L.M., Sharipova M.R. (2017). *Bacillus subtilis* strains with antifungal activity against the phytopathogenic fungi. Agric. Sci..

[87] Stein T. (2005). *Bacillus subtilis* antibiotics: Structures, syntheses and specific functions. Mol. Microbiol..

[88] Kloepper J.W., Gutierrez Estrada A., McInroy J.A. (2009). Photoperiod regulates elicitation of growth promotion but not induced resistance by plant growth promoting rhizobacteria. Can. J. Microbiol..

[89] Duffy B., Schouten A., Raaijmakers J.M. (2003). Pathogen selfdefense: Mechanisms to counteract microbial antagonism. Annu. Rev. Phytopathol..

[90] Chen X.-H., Scholz R., Borriss M., Junge H., Mögel G., Kunz S., Borris R. (2009). Difficidin and bacilysin produced by plant-associated *Bacillus amyloliquefaciens* are efficient in controlling fire blight disease. J. Biotechnol..

[91] Ongena M., Jourdan E., Adam A., Paquot M., Brans A., Joris B., Arpigny J.L., Thonart P. (2007). Surfactin and fengycin lipopeptides of *Bacillus subtilis* as elicitors of induced systemic resistance in plants. Environ. Microbiol..

[92] Ongena M., Henry G., Thonart P., Gisi U., Chet I., Gullino M. (2010). The role of cyclic lipopeptides in the biocontrol activity of *Bacillus subtilis*. Recent Developments in Management of Plant Diseases (Plant Pathology in the 21st Century).

[93] Ahmad Z., Wu J., Chen L., Dong W. (2017). Isolated *Bacillus subtilis* strain 330-2 and its antagonistic genes identified by the removing PCR. Sci. Rep..

[94] Loeffler W., Kratzer W., Kremer S., Kugler M., Petersen F., Jung G., Rapp C., Tschen J.S.M. (1990). Gegen pilze wirksame antibiotika der *Bacillus subtilis*-gruppe. Forum Mikrobiol..

[95] Arrebola E., Jacobs R., Korsten L. (2010). Iturin A is the principal inhibitor in the biocontrol activity of *Bacillus amyloliquefaciens* PPCB004 against postharvest fungal pathogens. J. Appl. Microbiol..

[96] Raaijmakers J.M., De Bruijn I., Nybroe O., Ongena M. (2010). Natural functions of lipopeptides from *Bacillus* and *Pseudomonas*: More than surfactants and antibiotics. FEMS Microbiol. Rev..

[97] Mora I., Cabrefiga J., Montesinos E. (2011). Antimicrobial peptide genes in *Bacillus* strains from plant environments. Int. Microbiol..

[98] Ongena M., Jacques P., Touré Y., Destain J., Jabrane A., Thonart P. (2005). Involvement of fengycin-type lipopeptides in the multifaceted biocontrol potential of *Bacillus subtilis*. Appl. Microbiol. Biotechnol..

[99] Luo C., Liu X., Zhou H., Wang X., Chen Z. (2015). Nonribosomal peptide synthase gene clusters for lipopeptide biosynthesis in *Bacillus subtilis* 916 and their phenotypic functions. Appl. Environ. Microbiol..

[100] Cho S.J., Lee S.K., Cha B.J., Kim Y.H., Shin K.-S. (2003). Detection and characterization of the *Gloeosporium gloeosporioides* growth inhibitory compound iturin A from *Bacillus subtilis* strain KS03. FEMS Microbiol. Lett..

[101] Romero D., de Vicente A., Rakotoaly R.H., Dufour S.E., Veening J.-W., Arrebola E., Cazorla F.M., Kuipers O.P., Paquot M., PérezGarcía A. (2007). The iturin and fengycin families of lipopeptides are key factors in antagonism of *Bacillus subtilis* toward *Podosphaera fusca*. Mol. Plant Microbe Interact..

[102] Jones T.S. (1949). Chemical evidence for the multiplicity of the antibiotics produced by Bacillus polymyxa. Ann. N. Y. Acad. Sci..

[103] Falardeau J., Wise C., Novitsky L., Avis T.J. (2013). Ecological and mechanistic insights into the direct and indirect antimicrobial properties of *Bacillus subtilis* lipopeptides on plant pathogens. J. Chem. Ecol..

[104] Jasim B., Sreelakshmi K.S., Mathew J., Radhakrishnan E.K. (2016). Surfactin, iturin and fengycin biosynthesis by endophytic *Bacillus* sp. from Bacopa monnieri. Microb. Ecol..

[105] Peypoux F., Bonmatin J.M., Wallach J. (1999). Recent trends in the biochemistry of surfactin. Appl. Microbiol. Biotechnol..

[106] Mihalache G., Balaes T., Gostin I., Stefan M., Coutte F., Krier F. (2017). Lipopeptides produced by Bacillus subtilis as new biocontrol products against fusariosis in ornamental plants. Environ. Sci. Pollut. Res..

[107] Aranda F.J., Teruel J.A., Ortiz A. (2005). Further aspects on the hemolytic activity of the antibiotic lipopeptide iturin A. Biochim. Biophys. Acta.

[108] Dixon R.A., Achnine L., Kota P., Liu C.J., Reddy M.S., Wang L. (2002). The phenylpropanoid pathway and plant defense—A genomics perspective. Mol. Plant Pathol..

[109] Rahman A., Uddin W., Wenner N.G. (2015). Induced systemic resistance responses in perennial ryegrass against Magnaporthe oryzae elicited by semi-purified surfactin lipopeptides and live cells of Bacillus amyloliquefaciens. Mol. Plant Pathol..

[110] Kong H.G., Kim J.C., Choi G.J., Lee K.Y., Kim H.J., Hwang E.C., Moon B.-J., Lee S.W. (2012). Production of surfactin and iturin by *Bacillus licheniformis* N1 responsible for plant disease control activity. Plant Pathol. J..

[111] Waewthongrak W., Pisuchpen S., Leelasuphakul W. (2015). Effect of *Bacillus subtilis* and chitosan applications on green mold (*Penicilium digitatum* Sacc.) decay in citrus fruit. Postharvest Biol. Technol..

[112] Krebs B., Ockhardt A., Hoeding B., Bendzko P., Maximov J., Etzel W. (1996). Cyclic Peptides from *Bacillus amyloliquefaciens* Useful Antimycotics, Antivirals, Fungicides, Nematicides etc. German Patent.

[113] Banat I.M., Makkar R.S., Cameotra S.S. (2000). Potential commercial applications of microbial surfactants. Appl. Microbiol. Biotechnol..

[114] Marrone P.G. (2002). An effective biofungicide with novel modes of action. Pestic. Outlook.

[115] Singh P., Cameotra S.S. (2004). Enhancement of metal bioremediation by use of microbial surfactants. Biochem. Biophys. Res. Commun..

[116] Gueldner R.C., Reilly C.C., Pussey P.L., Costello C.E., Arrendale R.F., Cox R.H., Himmelsbach D.S., Crumley F.G., Culter H.G. (1988). Isolation and identification of iturins as antifungal peptides in biological control of peach brown rot with *Bacillus subtilis*. J. Agric. Food Chem..

[117] Stein T., Entian K.-D. (2002). Maturation of the lantibiotic subtilin: Matrix-assisted laser desorption/ionization time-of-flight mass spectrometry to monitor precursors and their proteolytic processing in crude bacterial cultures. Rapid Commun. Mass Spectrom..

[118] Phister T.G., O’Sullivan D.J., McKay L.L. (2004). Identification of bacilysin, chlorotetaine, and iturin A produced by *Bacillus* sp. strain CS93 isolated from pozol, a Mexican fermented maize dough. Appl. Environ. Microbiol..

[119] Bongers R.S., Veening J.-W., Van Wieringen M., Kuipers O.P., Kleerebezem M. (2005). Development and characterization of a subtilin-regulated expression system in *Bacillus subtilis*: Strict control of gene expression by addition of subtilin. Appl. Environ. Microbiol..

[120] Rajavel M., Mitra A., Gopal B. (2009). Role of *Bacillus subtilis* BacB in the synthesis of bacilysin. J. Biol. Chem..

[121] Lee H., Kim H.-Y. (2011). Lantibiotics, class I bacteriocins from the genus *Bacillus*. J. Microbiol. Biotechnol..

[122] Chung S., Kong H., Buyer J.S., Lakshman D.K., Lydon J., Kim S.-D., Roberts D.P. (2008). Isolation and partial characterization of *Bacillus subtilis* ME488 for suppression of soilborne pathogens of cucumber and pepper. Appl. Microbiol. Biotechnol..

[123] Joshi R., McSpadden-Gardener B.B. (2006). Identification and characterization of novel genetic markers associated with biological control activities in *Bacillus subtilis*. Phytopathology.

[124] González-Sánchez M.A., Pérez-Jimenez R.M., Pliego C., Ramos C., de Vicente A., Cazorla F.M. (2010). Biocontrol bacteria selected by a direct plant protection strategy against avocado white root rot show antagonism as a prevalent trait. J. Appl. Microbiol..

[125] Haggag W.M. (2008). Isolation of bioactive antibiotic peptides from *Bacillus brevis* and *Bacillus polymyxa* against *Botrytis grey* mold in strawberry. Arch. Phytopathol. Plant Protect..

[126] Gong Q., Zhang C., Lu F., Zhao H., Bie X., Lu Z. (2013). Identification of bacillomycin D from *Bacillus subtilis* fmbJ and its inhibition effects against *Aspergillus flavus*. Food Control.

[127] Bull C.T., Wadsworth M.L.K., Sorenson K.N., Takemoto J., Austin R., Smilanick J.L. (1998). Syringomycin E produced by biological agents controls green mold on lemons. Biol. Control.

[128] Bais H.P., Fall R., Vivanco J.M. (2004). Biocontrol of *Bacillus subtilis* against infection of arabidopsis roots by *Pseudomonas syringae* is facilitated by biofilm formation and surfactin production. Plant Physiol..

[129] Asaka O., Shoda M. (1996). Biocontrol of *Rhizoctonia solani* damping-off of tomato with *Bacillus subtilis* RB14. Appl. Environ. Microbiol..

[130] Kajimura Y., Sugiayama M., Kaneda M. (1995). Bacillopeptins, new cyclic lipopeptide antibiotics from *Bacillus subtilis* FR-2. J. Antibiot..

[131] Yánez-Mendizábal V., Usall J., Viñas I., Casals C., Marín S., Solsona C., Teixidó N. (2011). Potential of a new strain of *Bacillus subtilis* CPA-8 to control the major postharvest diseases of fruit. Biocontrol Sci. Technol..

[132] Singh D., Sharma R.R., Prasad D. (2009). Postharvest diseases of fruit and vegetables and their management. Sustainable Pest Management.

[133] Rodrigues L., Banat I.M., Teixeira J., Oliveira R. (2006). Biosurfactants: Potential applications in medicine. J. Antimicrob. Chemother..

[134] Bakker P.A.H.M., Pieterse C.M.J., Van Loon L.C. (2007). Induced systemic resistance by fluorescent *Pseudomonas* spp.. Phytopathology.

[135] Lorito M., Harman G.E., Hayes C.K., Broadway R.M., Trosomo A., Woo S.L., Di-Pietro A. (1993). Chitolytic enzymes produced by *Trichoderma harzianum*: Antifungal activity of purified endochitinase and chitobiase. Phytopathology.

[136] Chernin L., Chet I., Burns R.G., Dick R.P. (2002). Microbial enzymes in the biocontrol of plant pathogens and pests. Enzymes in the Environment: Activity, Ecology, and Applications.

[137] Sharma R.R., Singh D., Singh R. (2009). Biological control of postharvest diseases of fruits and vegetables by microbial antagonists: A review. Biol. Control.

[138] Aktuganov G.E., Galimzyanova N.F., Melent’ev A.I., Kuzmina L.Y. (2007). Extracellular hydrolases of strain *Bacillus* sp. 739 and their involvement in the lysis of micromycete cell walls. Microbiology (Mikrobiologiya).

[139] Kavitha K., Nakkeeran S., Chandrasekar G. (2012). Rhizobacterial mediated induction of defense enzymes to enhance the resistance of turmeric (*Curcuma longa* L.) to *Pythium aphanidermatum* causing rhizome rot. Arch. Phytopathol. Plant Protect..

[140] Torres M.A. (2010). ROS in biotic interactions. Physiol. Plant..

[141] Sewelam N., Kazan K., Thomas Hall S.R., Kidd B.N., Manners J.M., Schenk P.M. (2013). Ethylene response factor 6 is a regulator of reactive oxygen species signaling in Arabidopsis. PLoS ONE.

[142] Maksimov I.V., Abisgildina R.R., Yusupova Z.R., Khairullin R.M. (2010). Effect of *Bacillus subtilis* 26D on the hydrogen peroxide level and peroxidase activity in spring wheat plants. Agrochemistry.

[143] Schoonbeek H.J., Jacquat Bovet A.C., Mascher F., Metraux J.P. (2007). Oxalate degrading bacteria can protect Arabidopsis thaliana and crop plants against *Botrytis cinerea*. Mol. Plant Microbe Interact..

[144] Kumar P., Dubey R.C., Maheshwari D.K. (2012). *Bacillus* strains isolated from rhizosphere showed plant growth promoting and antagonistic activity against phytopathogens. Microbiol. Res..

[145] Swain P., Nayak S.K., Nanda P.K., Dash S. (2008). Biological effects of bacterial lipopolysaccharide (endotoxin) in fish: A review. Fish Shellfish Immunol..

[146] Janisiewicz W.J., Korsten L. (2002). Biological control of postharvest diseases of fruits. Annu. Rev. Phytopathol..

[147] Pieterse C.M., Zamioudis C., Berendsen R.L., Weller D.M., van Wees S.C., Bakker P.A. (2014). Induced systemic resistance by beneficial microbes. Annu. Rev. Phytopathol..

[148] Pieterse C.M.J., Van der Does D., Zamioudis C., Leon Reyes A., van Wees S.C.M. (2012). Hormonal modulation of plant immunity. Annu. Rev. Cell Dev. Biol..

[149] Dodd I.C., Zinovkina N.Y., Safronova V.I., Belimov A.A. (2010). Rhizobacterial mediation of plant hormone status. Ann. Appl. Biol..

[150] Dobbelaere S., Vanderleyden J., Okon Y. (2003). Plant growthpromoting effects of diazotrophs in the rhizosphere. Crit. Rev. Plant Sci..

[151] Sivasakthi S., Kanchana D., Usharani G., Saranraj P. (2013). Production of plant growth promoting substance by Pseudomonas fluorescens and Bacillus subtilis isolates from paddy rhizosphere soil of Cuddalore district, Tamil Nadu, India. Int. J. Microbiol. Res..

[152] Kudoyarova G.R., Melentiev A.I., Martynenko E.V., Timergalina L.N., Arkhipova T.N., Shendel G.V., Kuz’mina L.Y., Dodd I.C., Veselov S.Y. (2014). Cytokinin producing bacteria stimulate amino acid deposition by wheat roots. Plant Physiol. Biochem..

[153] Belimov A.A., Dodd I.C., Safronova V.I., Dumova V.A., Shaposhnikov A.I., Ladatko A.G., Davies W.J. (2014). Abscisic acid metabolizing rhizobacteria decrease ABA concentrations in planta and alter plant growth. Plant Physiol. Biochem..

[154] Cohen A.C., Travaglia C.N., Bottini R., Piccoli P.N. (2009). Participation of abscisic acid and gibberellins produced by endophytic *Azospirillum* in the alleviation of drought effects in maize. Botany.

[155] Zhang H., Kim M.S., Krishnamachari V., Payton P., Sun Y., Grimson M., Farag M.A., Ryu C.M., Allen R., Melo I.S. (2007). Rhizobacterial volatile emissions regulate auxin homeostasis and cell expansion in Arabidopsis. Planta.

[156] Zhang H., Xie X., Kim M.S., Kornyeyev D.A., Holaday S., Paré P.W. (2008). Soil bacteria augment Arabidopsis photosynthesis by decreasing glucose sensing and abscisic acid levels in planta. Plant J..

[157] Pertry I., Václavíková K., Depuydt S., Galuszka P., Spíchal L., Temmerman W., Stes E., Schmülling T., Kakimoto T., van Montagu M.C.E. (2006). Identification of *Rhodococcus fascians* cytokinins and their modus operandi to reshape the plant. Proc. Natl. Acad. Sci. USA.

[158] Garcia-Gutierrez L., Romero D., Zeriouh H., Cazorla F.M., Torés J.A., de Vicente A., Pérez-García A. (2012). Isolation and selection of plant growth-promoting rhizobacteria as inducers of systemic resistance in melon. Plant Soil.

[159] Spadaro D., Gullino M.L. (2004). State of the art and future prospects of the biological control of postharvest fruit diseases. Int. J. Food Microbiol..

[160] Razzaq K., Khan A.S., Malik A.U., Shahid M. (2013). Ripening period influences fruit softening and antioxidative system of ‘Samar Bahisht Chaunsa’ mango. Sci. Hortic..

[161] Li T., Yun Z., Zhang D.D., Yang C.W., Zhu H., Jiang Y.M., Duan X.W. (2015). Proteomic analysis of differentially expressed proteins involved in ethylene-induced chilling tolerance in harvested banana fruit. Front. Plant Sci..

[162] Belimov A.A., Dodd I.C., Hontzeas N., Theobald J.C., Safronova V.I., Davies W.J. (2009). Rhizosphere bacteria containing ACC deaminase increase yield of plants grown in drying soil via both local and systemic hormone signaling. New Phytol..

[163] Gamalero E., Glick B.R. (2015). Bacterial modulation of plant ethylene levels. Plant Physiol..

[164] Farace G., Fernandez O., Jacquens L., Coutte F., Krier F., Jacques P., Clément C., Barka E.A., Jacquard C., Dorey S. (2015). Cyclic lipopeptides from Bacillus subtilis activate distinct patterns of defense responses in grapevine. Mol. Plant Pathol..

[165] Sarosh B.R., Danielsson J., Meijer J. (2009). Transcript profiling of oilseed rape (*Brassica napus*) primed for biocontrol differentiate genes involved in microbial interactions with beneficial *Bacillus amyloliquefaciens* from pathogenic *Botrytis cinerea*. Plant Mol. Biol..

[166] Chowdhury S.P., Uhl J., Grosch R., Alquéres S., Pittroff S., Dietel K., Schmitt-Kopplin P., Borriss R., Hartmann A. (2015). Cyclic lipopeptides of *Bacillus amyloliquefaciens* subsp. plantarum colonizing the lettuce rhizosphere enhance plant defense responses toward the bottom rot rathogen *Rhizoctonia solani*. Mol. Plant Microbe Interact..

[167] Chandler S., Van Hese N., Coutte F., Jacques P., Hofte M., De Vleesschauwer D. (2015). Role of cyclic lipopeptides produced by *Bacillus subtilis* in mounting induced immunity in rice (*Oryza sativa* L.). Physiol. Mol. Plant Pathol..

[168] Zhang S., Jiang W., Li J., Meng L., Cao X., Hu J., Liu Y., Chen J., Sha C. (2016). Whole genome shotgun sequence of Bacillus amyloliquefaciens TF28, a biocontrol entophytic bacterium. Stand. Genomic Sci..

[169] Rodov V., Ben-Yehoshua S., Fang D.Q., D’hallewin G., Castia T. (1994). Accumulation of phytoalexins scoparone and scopoletin in citrus fruits subjected to various postharvest treatments. Acta Hortic..

[170] Arras G. (1996). Mode of action of an isolate of *Candida famata* in biological control of *Penicillium digitatum* in orange fruits. Postharvest Biol. Technol..

[171] Ahn I.P., Lee S.W., Kim M.G., Park S.R., Hwang D.J., Bae S.C. (2011). Priming by rhizobacterium protects tomato plants from biotrophic and necrotrophic pathogen infections through multiple defense mechanisms. Mol. Cells.

[172] Nakkeeran S., Kavitha K., Chandrasekar G., Renukadevi P., Fernando W.G.D. (2006). Induction of plant defense compounds by *Pseudomonas chlororaphis* PA23 and Bacillus subtilis BSCBE4 in controlling damping-off of hot pepper caused by *Pythium aphanidermatum*. Biocontrol Sci. Technol..

[173] Thilagavathi R., Saravanakumar D., Ragupathi N., Samiyappan R. (2007). A combination of biocontrol agents improves the management of dry root rot (*Macrophomina phaseolina*) in greengram. Phytopathol. Mediterr..

[174] Da K., Nowak J., Flinn B. (2012). Potato cytosine methylation and gene expression changes induced by a beneficial bacterial endophyte, *Burkholderia phytofirmans* strain PsJN. Plant Physiol. Biochem..

[175] Coates L., Johnson G., Dale M., Brown J.F., Ogle H.J. (1997). Postharvest diseases of fruit and vegetables. Plant Pathogens and Plant Diseases.

[176] Ippolito A., Nigro F. (2000). Impact of preharvest application of biological control agents on postharvest diseases of fresh fruit and vegetables. Crop Prot..

[177] Ippolito A., Schena L., Pentimone I., Nigro F. (2005). Control of postharvest rots of sweet cherries by pre- and postharvest applications of *Aureobasidium pullulans* in combination with calcium chloride or sodium bicarbonate. Postharvest Biol. Technol..

[178] Irtwange S.V. (2006). Application of biological control agents in pre- and postharvest operations. Agric. Eng. Int. CIGR J..

[179] Leibinger W., Breuker B., Hahn M., Mendgen K. (1997). Control of postharvest pathogens and colonization of the apple surface by antagonistic microorganisms in the field. Phytopathology.

[180] Narayanasamy P. (2005). Ecology of Postharvest Microbial Pathogens. Postharvest Pathogens and Disease Management.

[181] Korsten L., Bezuidenhout J.J., Kotzé J.M. (1988). Biological control of postharvest diseases of avocado. South African Avocado Growers’ Association Yearbook.

[182] Barkai-Golan R. (2001). Postharvest diseases of fruit and vegetables. Development and Control.

[183] Droby S. (2006). Improving quality and safety of fresh fruit and vegetables after harvest by the use of biocontrol agents and natural materials. Acta Hortic..

[184] Pusey P.L., Hotchkiss M.W., Dulmage H.T., Banumgardner R.A., Zehr E.I. (1988). Pilot tests for commercial production and application of *Bacillus subtilis* (B-3) for postharvest control of peach brown rot. Plant Dis..

[185] Anon (2010). AgraQuest Introduces New Soil Fungicide for Potatoes and Other Crops. http://agraquest.com/news/2010/01/agraquest-introduces-newsoil-fungicide-for-potatoes-and-other-crops/.

[186] Walton D. (2002). New Zealand Agrichemical Manual.

